# Gentamicin Rapidly Inhibits Mitochondrial Metabolism in High-Frequency Cochlear Outer Hair Cells

**DOI:** 10.1371/journal.pone.0038471

**Published:** 2012-06-08

**Authors:** Heather C. Jensen-Smith, Richard Hallworth, Michael G. Nichols

**Affiliations:** 1 Department of Biomedical Sciences, Creighton University, Omaha, Nebraska, United States of America; 2 Department of Physics, Creighton University, Omaha, Nebraska, United States of America; University of South Alabama, United States of America

## Abstract

Aminoglycosides (AG), including gentamicin (GM), are the most frequently used antibiotics in the world and are proposed to cause irreversible cochlear damage and hearing loss (HL) in 1/4 of the patients receiving these life-saving drugs. Akin to the results of AG ototoxicity studies, high-frequency, basal turn outer hair cells (OHCs) preferentially succumb to multiple HL pathologies while inner hair cells (IHCs) are much more resilient. To determine if endogenous differences in IHC and OHC mitochondrial metabolism dictate differential sensitivities to AG-induced HL, IHC- and OHC-specific changes in mitochondrial reduced nicotinamide adenine dinucleotide (NADH) fluorescence during acute (1 h) GM treatment were compared. GM-mediated decreases in NADH fluorescence and succinate dehydrogenase activity were observed shortly after GM application. High-frequency basal turn OHCs were found to be metabolically biased to rapidly respond to alterations in their microenvironment including GM and elevated glucose exposures. These metabolic biases may predispose high-frequency OHCs to preferentially produce cell-damaging reactive oxygen species during traumatic challenge. Noise-induced and age-related HL pathologies share key characteristics with AG ototoxicity, including preferential OHC loss and reactive oxygen species production. Data from this report highlight the need to address the role of mitochondrial metabolism in regulating AG ototoxicity and the need to illuminate how fundamental differences in IHC and OHC metabolism may dictate differences in HC fate during multiple HL pathologies.

## Introduction

According to the World Health Organization, deafness and hearing impairments affect more than 278 million individuals, indicating hearing loss (HL) is the most frequent sensory deficit in global populations. Aminoglycoside (AG) antibiotics are frequently used to treat life-threatening gram-negative infections but their clinical utility is limited due to nephrotoxicity and ototoxicity [1]. Unlike AG-induced nephrotoxicity, AG-induced ototoxicity is irreversible and proposed to cause HL and/or deafness in 25% of patients receiving these life-saving antibiotics [1], [2]. Of the two types of cochlear sensory hair cells, outer hair cells (OHCs) reliably succumb to a barrage of AG-triggered pro-apoptotic signals, while inner hair cells (IHCs) display a truncated pro-apoptotic signaling response and greater survival, relative to OHCs [3]–[6]. Additionally, when compared to apical turn, low-frequency processing OHCs, basal turn, high-frequency processing OHCs are preferentially damaged.

Although there are numerous causes of HL and deafness, reactive oxygen species (ROS) are now well-known instigators of multiple HL pathologies including: aminoglycoside (AG)-induced ototoxicity (recent review: [7]), noise-induced (NIHL, [8], [9]), and age-related HL (ARHL, review: [10]). ROS are normal byproducts of ATP synthesis that can rise to lethal levels when mitochondrial metabolism is perturbed. AGs have been shown to enter inner hair cells and outer hair cells (I/OHCs) at the apical pole and preferentially accumulate in mitochondria [11]–[13]. Gentamicin (GM), a representative AG antibiotic, has also been shown to directly inhibit protein synthesis in human mitochondrial ribosomes [14], [15] and trigger mitochondrial permeability transition pore opening in cochlear HCs [16]. Likewise, mitochondrial mutations are commonly associated with sensorineural HL [17]–[20] and in some individuals a profound susceptibility to AG-induced HL [14], [21]–[24]. Others have also shown that cellular ATP concentration can dictate commitment to apoptotic or necrotic cell fates for multiple cell types [25]–[27]. For cochlear I/OHCs, succinate dehydrogenase (SDH) activity, a mitochondrial enzyme, is a key arbitrator of HC fate during acoustic trauma and exposure to various ototoxic agents [28]–[31]. As such, intrinsic differences in I/OHC mitochondrial metabolism may explain why high-frequency OHCs are profoundly sensitive to mitochondrial-mediated damage during various cochlear pathologies.

Mitochondrial metabolism couples oxidative phosphorylation (the electron transport chain) to the generation of ATP. During oxidative phosphorylation, free energy released from glucose oxidation is harnessed by transferring electrons from the reducing agents NADH, FADH_2_ and succinate through a series of electron carriers, including ubiquinone, in the inner mitochondrial membrane. NADH, the primary electron donor/reducing agent, is fluorescent (Fl) when reduced (NADH) and non-fluorescent when oxidized (NAD^+^). NADH Fl represents the net activities of two opposing processes; Krebs cycle-mediated NADH reduction/production (increases NADH Fl, NADH) and electron transport chain-mediated NADH oxidation/utilization (decreases NADH Fl by increasing NAD^+^). If metabolic demands increase, the NADH/NAD^+^ ratio will, at least temporarily, decrease resulting in a reduction in NADH Fl intensity. As such, mitochondrial function can be evaluated by measuring real-time changes in NADH Fl in intact cells [32], [33]. Indeed, two-photon confocal imaging of NADH Fl was recently used to observe *real-time* changes in mitochondrial metabolism in living isolated cochlear preparations [34], [35]. As indicated by a decrease in NADH Fl, these studies revealed GM rapidly altered OHC, but not IHC, mitochondrial metabolism. These results also suggested a GM-induced decrease in ATP synthesis and presumably OHC viability, occurred within minutes of GM exposure.

It is important to note that the Fl spectra of NADH and nicotinamide adenine dinucleotide phosphate (NADPH) are indistinguishable. NADPH is a reducing agent for lipid and amino acid synthesis that is also capable of regenerating cellular antioxidants (glutathione) and triggering free-radical production in immune cells. As described in a previous study of NAD(P)H metabolism in cochlear HCs, the contribution of NADH and NADPH to the total NAD(P)H signal can be reasonably determined by examining changes in NAD(P)H Fl during treatment with the metabolic uncoupler FCCP and the metabolic poison sodium cyanide [35]. By measuring NADH and flavoprotein Fl during the aforementioned metabolic perturbations, Tiede at al. confirmed NADH, not NADPH Fl, prevails in cochlear I/OHCs. In light of the fact that NaCN and FCCP specifically alter mitochondrial metabolism and that the sum of the relative oxidation and reduction percentages for cochlear HCs always totaled 100%, the observed changes in I/OHC NADH indicated NADH Fl predominantly originated from mitochondrial sources. Given these findings, the NADH Fl described in this report is considered to be principally mitochondrial and comparatively free of NADPH.

The current report uses acutely-cultured perinatal cochlear explants to further probe the nature of the rapid, GM-induced decrease in mitochondrial metabolism (the GM NADH effect) and to determine if OHC-specific decreases in the NADH Fl are due to increased NADH oxidation or decreased Krebs cycle-mediated NADH reduction. In the freshly-dissected adult cochlea imaging technique previously used [34], [35], calcified bone prevented transmitted light imaging to verify HC morphology and viability. By using acutely-cultured perinatal cochlear explants, transmitted light imaging of cellular morphology and HC viability can be obtained throughout each experiment. During the acute (24 h) culturing period, viable HCs will maintain organized stereocilia, non-granular cytoplasm and will appropriately restrict cellular swelling, while traumatized HCs will show morphological abnormalities, including splayed stereociliary bundles, plasma membrane blebbing, swelling, and granular inclusions in the cytoplasm. Finally, freshly-dissected cochlear preparations remain viable for no more than 2 h after opening of the cochlea. Cultured preparations, on the other hand, can be maintained for hours to over one week [36] permitting reporter dye uptake for functional analyses, as well as time to apply and evaluate the effects of putative I/OHC-saving treatments.

Given that basal turn, high-frequency OHCs are reliably lost during GM treatment, identification of the process(s) responsible for the rapid GM-induced decrease in NADH Fl is critical for developing new HL prevention strategies. By transitioning to the acutely cultured technique, a host of new studies examining the metabolic mechanisms mediating GM ototoxicity are possible. Furthermore, by uncovering fundamental metabolic differences between cochlear I/OHCs and the mechanisms mediating OHC-specific decreases in the NADH Fl, this report provides critical data for the development of additional HL prevention and treatment strategies targeting multiple HL pathologies.

## Results

### Endogenous Differences in Cochlear IHC and OHC Metabolism

**Figure 1:**
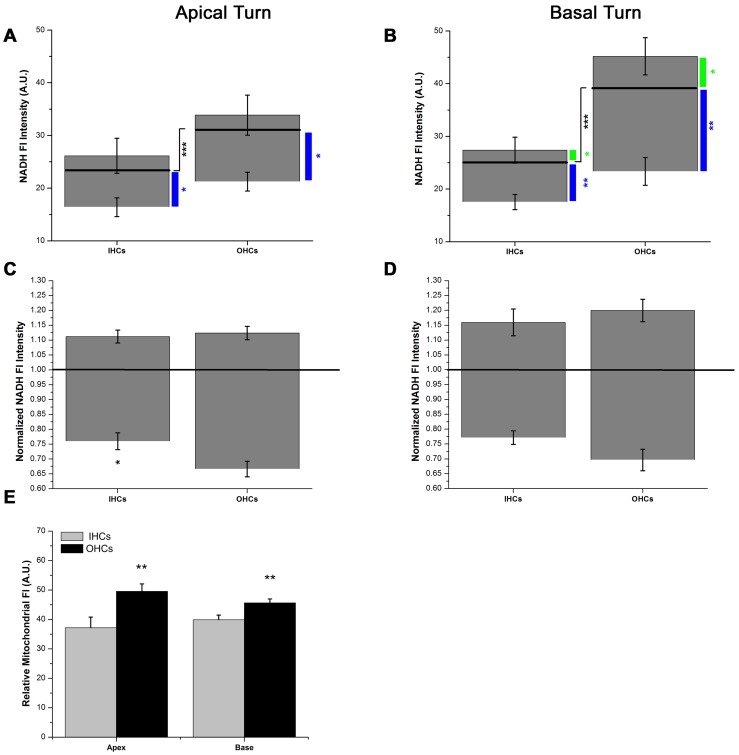
Endogenous differences in I/OHC NADH metabolism. A) Steady-state, endogenous NADH Fl in apical turn OHCs is greater than apical turn IHCs (black line, t(62) = 4.31, p<0.001, n = 32). Absolute increases in NADH Fl during maximum NADH reduction (NaCN, n = 8) were similar for apical I/OHCs while maximum NADH oxidation (FCCP, n = 6) caused a greater decrease in NADH Fl in apical OHCs than in apical IHCs (t(10)** = **4.49, p<0.01). B) Steady-state, endogenous NADH Fl in basal turn OHCs is greater than basal turn IHCs (black line, t(64) = 4.15, p<0.001, n = 33). C) Normalized increases in NADH Fl during maximum NADH reduction (NaCN, n = 8) were similar for apical turn I/OHCs while decreases in NADH Fl during maximum NADH oxidation (FCCP, n = 6) were greater in OHCs than in IHCs (t(10) = 2.22, p<0.05). D) Normalized increases in NADH Fl during maximum NADH reduction and decreases in NADH Fl during maximum NADH oxidation were similar in basal turn I/OHCs. E) Relative differences in Mitotracker Red Fl intensity indicated OHCs in both cochlear location contained more functional mitochondria than IHCs at each location (n_apex_ = 7, n_base_ = 7). * = p<0.05, ** = p<0.01, *** = p<0.001.

Recall that baseline NADH Fl represents the opposing actions of Krebs cycle-mediated NADH reduction/production and electron transport-mediated NADH oxidation/utilization. Putative intrinsic differences in I/OHC mitochondrial metabolism were assessed by examining baseline NADH Fl intensities in low- and high-frequency I/OHCs. IHC and OHC endogenous NADH Fl intensities were significantly different (Fig. 1A,B, black bar). Baseline, endogenous NADH Fl in apical turn OHCs was greater than that of apical turn IHCs (t(62) = 4.31, p<0.001). The greatest difference in endogenous NADH Fl intensity occurred between basal turn IHCs (25.19±0.93) and OHCs (39.72±1.56, t(64) = 8.11, p<0.001).

Next, NADH was maximally reduced using 10 µM NaCN (10 min) or maximally oxidized using 10 µM carbonylcyanide-p-trifluoromethoxyphenylhydrazone (FCCP, 10 min) [35]. After NaCN application, the absolute increase in NADH Fl was similar for apical turn I/OHCs (Fig. 1A). In high-frequency regions of the cochlea, OHCs displayed a larger increase in NADH Fl than IHCs (Fig. 1B, t(14) = 1.86, p<0.05). After FCCP application, decreases in NADH Fl were greater in OHCs than in IHCs in both low- and high-frequency regions of the cochlea (t(10) = 2.7, p<0.01, t(10) = 4.49, p<0.01), respectively). Given that baseline differences in NADH Fl exist between I/OHCs, a relative redox state scale was calculated for apical and basal turn I/OHCs during maximum NADH reduction and oxidation (Fig. 1C,D). The relative redox scale indicates NaCN-induced maximum NADH reduction is similar in low- and high-frequency I/OHCs. On the other hand, relative differences in FCCP-induced maximum NADH oxidation were significant between apical I/OHCs (t(10) = 2.22, p<0.05) and approached significance for basal turn I/OHCs (p = 0.054).

Differences in functional mitochondrial densities were determined by examining relative differences in Mitotracker Red CM-H_2_XRos Fl intensity in high- and low-frequency I/OHCs (Fig. 1E). In both apical and basal regions of the cochlea, OHC mitochondrial densities were greater than IHC mitochondrial densities (t(7) = 2.80, p<0.01, t(7) = 2.77, p<0.01, respectively).

### GM Rapidly Decreases NADH Fluorescence (GM NADH Effect)

**Figure 2:**
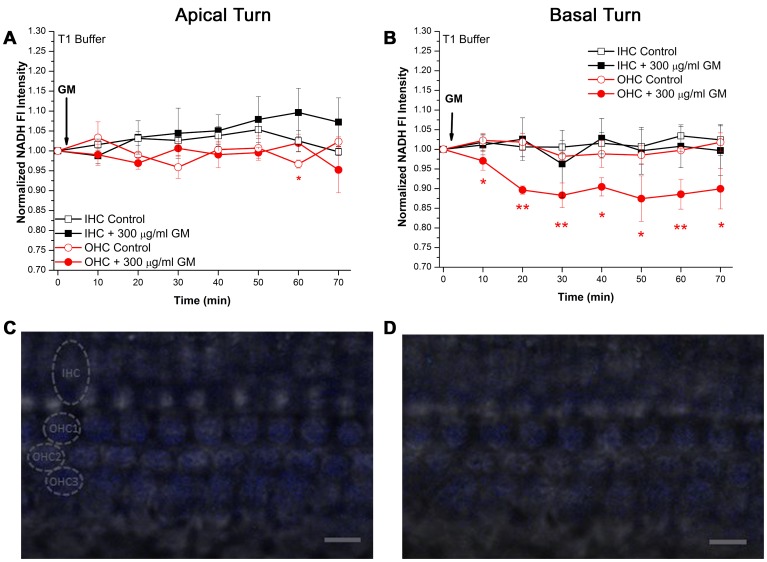
Acute GM exposure decreased NADH Fl in basal turn OHCs bathed in T1 imaging buffer. A) I/OHCs in apical, low-frequency regions of the cochlea do not show significant alterations in NADH Fl during acute GM exposure (n_GM_ = 5, n_Cont_ = 4). A transient difference was observed in OHCs at 60 min (t(7) = 1.96, p<0.05). B) Although basal turn, high-frequency IHCs were unaltered by GM, basal turn OHCs displayed a significant decrease in NADH Fl within 10 min of GM exposure (t(7) = 2.0, p<0.05, n_GM_ = 5, n_Cont_ = 4). C) Representative image of NADH Fl intensity in basal turn I/OHCs before and after (D) GM exposure. The location of the IHC row and OHC rows are indicated by circling an individual HC from each location. T1 imaging buffer contained 5 mM glucose. Scale bar = 10 µm. * = p<0.05, ** = p<0.01.

Akin to a previous experiment using freshly-dissected adult cochleae [34], 300 µg/ml GM rapidly decreased NADH Fl in cochlear OHCs housed in acutely-cultured, intact organ of Corti explants from postnatal day 6±1 day mice (Fig. 2). NADH Fl in apical turn, low-frequency I/OHCs was not significantly altered by acute GM treatment (Fig. 2A). Although a significant difference between control and GM-treated OHCs was observed at 60 min, this result was transient and failed to intimate any difference between control and GM-treated OHCs. In contrast to low-frequency, apical turn I/OHCs, basal turn, high-frequency OHCs displayed a significant decrease in NADH Fl intensity within 10 min of 300 µg/ml GM application (t(7) = 2.00, p<0.05, Fig. 2B). Basal turn OHCs maintained a 10–12% decrease in NADH Fl intensity throughout the GM exposure period while NADH Fl in basal turn IHCs remained unaltered.

In contrast to apical turn GM-exposed I/OHCs, which maintained similar NADH Fl intensities throughout the GM exposure period, NADH Fl in GM-exposed basal turn OHCs was significantly lower than in GM-exposed basal turn IHCs within 20 min (IHCs = 1.025±0.054, OHCs = 0.896±0.01, t(8) = 3.014, p<0.01). The significant suppression of NADH Fl in GM-exposed basal turn OHCs, relative to GM-exposed basal turn IHCs, remained after 1 h (IHCs = 0.997±0.063, OHCs = 0.900±0.051, t(8) = 1.897, p<0.05). Figures 2C and D represent basal turn I/OHC NADH Fl before and after GM-exposure, respectively.

### GM NADH Effect: NADH Oxidation Assay

**Figure 3:**
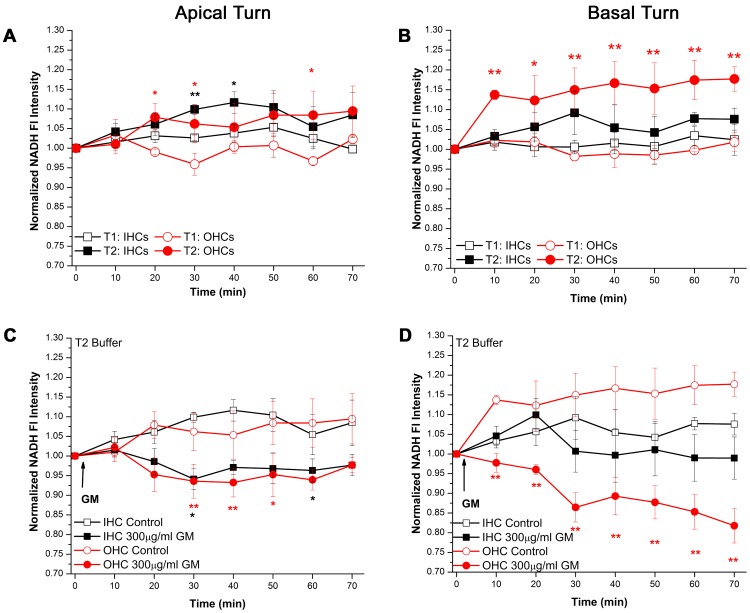
T2 buffer increased NADH reduction/production but failed to prevent the GM NADH effect. A) Apical turn I/OHCs display transient, significant increases in NADH Fl when bathed in T2 buffer (n_T1_ = 5, n_T2_ = 4). B) Basal turn OHCs display a significant and prolonged increase in NADH Fl in T2 buffer while basal turn IHCs do not (n_T1_ = 5, n_T2_ = 4). C) T2-bathed, apical turn I/OHCs display moderate decreases in NADH Fl during acute GM exposure (n_GM_ = 4, n_Cont_ = 5). D) T2-bathed basal turn OHCs, not basal turn IHCs, display significant and prolonged decreases in NADH Fl during GM exposure (n_GM_ = 5, n_Cont_ = 5). T2 imaging buffer contained 10 mM glucose, 3 mM glutamate and 2 mM pyruvate. * = p<0.05, ** = p<0.01.

If the GM NADH effect observed in basal turn OHCs was due to an increase in energetic demand, NADH oxidation would exceed NADH reduction resulting in a net decrease in NADH Fl. Therefore, if the concentration of reduced NADH in I/OHCs was significantly increased prior to GM application, the GM NADH effect ought to be diminished. As described in Materials and Methods, the baseline modified Tyrodes imaging buffer (T1), in which the GM NADH effect was initially observed (Fig. 2), contained 5 mM glucose. To increase NADH levels, a high-glucose (10 mM) Krebs cycle-substrate modified (3 mM glutamate, 2 mM pyruvate) imaging buffer (T2) was applied to the cochlear preparations. T2 buffer significantly increased NADH Fl in cochlear I/OHCs (Fig. 3A,B). Significant increases in NADH Fl intensity in apical turn I/OHCs were observed at multiple time points (Fig. 3A). A prolonged and large increase in NADH Fl intensity occurred in basal turn OHCs (Fig. 3B) within 10 min of T2 exposure (OHCs_T1_ = 1.023±0.017, OHCs_T2_ = 1.137±0.009, t(7) = 6.22, p<0.001). Basal turn OHCs maintained a ∼15% elevation in NADH Fl intensity throughout T2 exposure.

To determine if GM increases NADH oxidation (increased energetic demand), 300 µg/ml GM was applied to cochlear preparations pretreated (10–15 min) and subsequently maintained in T2 buffer. Despite the previously described T2-mediated increase in NADH (Fig. 3A, B), the GM NADH effect was still observed (Fig. 3C, D). When bathed in T2 buffer, apical turn I/OHCs displayed significant, yet transient decreases in NADH Fl 30 min after GM application (IHC_control_ = 1.099±0.013, IHC_GM_ = 0.941±0.027, OHC_control_ = 1.062±0.048, OHC_GM_ = 0.936±0.043, Fig. 3C). A significant and prolonged decrease in NADH Fl was observed in basal turn OHCs while basal turn IHCs did not display a significant decrease in NADH Fl (Fig. 3D). The basal turn GM NADH effect was substantial and prolonged, while the decrease in apical turn I/OHC NADH Fl was transient. Apical turn, GM-exposed I/OHCs maintained similar NADH Fl levels throughout the duration of the GM exposure, while NADH Fl levels in GM-exposed basal turn OHCs were significantly lower than GM-exposed IHCs within 10 min (OHCs_base_ = 0.978±0.024, IHCs_base_ = 1.046±0.024, t(8) = 1.99, p<0.05).

### GM NADH Effect: NADH Reduction Assay

Given that increased NADH oxidation did not appear to mediate the GM NADH effect in the previous experiment, the GM NADH effect may arise from a GM-induced decrease in NADH reduction/production. If so, NADH Fl should decrease when NADH production is dramatically decreased and NADH oxidation (conversion to non-Fl NAD^+^) is maintained.

**Figure 4:**
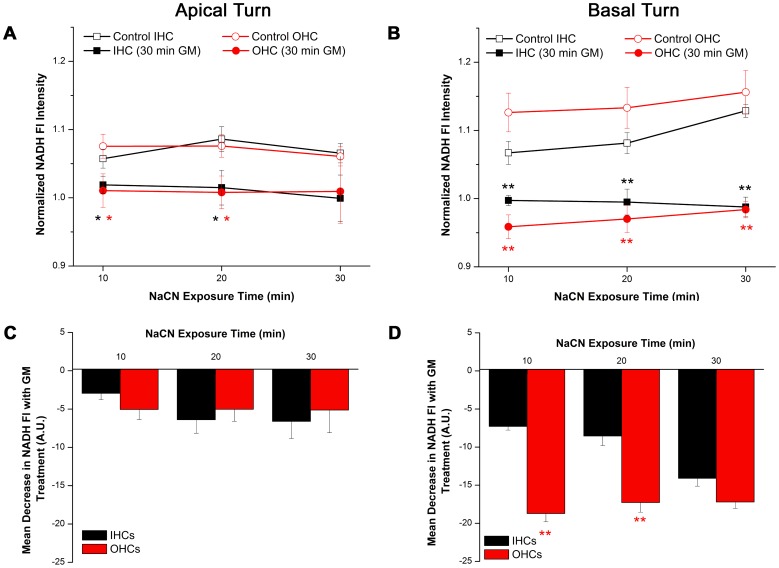
Acute GM exposure decreases NADH reduction/production capacity. A) NaCN-induced increases in NADH Fl in apical turn I/OHCs are significantly diminished when I/OHCs are pretreated with GM (300 µg/ml, n_GM_ = 8, n_Cont_ = 6). B) GM pretreatment (300 µg/ml) abolishes NaCN-induced increases in NADH Fl in basal turn I/OHCs (n_GM_ = 7, n_Cont_ = 6). C) Mean changes in NADH Fl between control and GM-exposed I/OHCs were similar in apical turn I/OHCs and D) greater in basal turn OHCs, relative to basal turn IHCs. Experiments conducted in T1 buffer. * = p<0.05, ** = p<0.01.

NADH production was assessed in control and GM-treated cochleae after NADH oxidation was inhibited with NaCN (10 µM). NaCN increases NADH Fl in cochlear I/OHCs bathed in T1 buffer (see controls, Fig. 4A, B). Similar to the dramatic and distinct T2-mediated increase in NADH Fl observed in basal turn OHCs, the largest NaCN-induced increase in NADH Fl was observed in basal turn OHCs (Fig. 4B). Specifically, NADH production capacity, measured as NADH Fl intensity when NADH oxidation is inhibited, was greater in basal turn OHCs, relative to apical turn OHCs (at 30 min: OHCs_apex_ = 1.06±0.014, OHCs_base_ = 1.156±0.032, t(7) = 2.514, p<0.05).

If GM limited NADH production, NaCN-mediated increases in NADH Fl would be similarly limited in GM pretreated I/OHCs. Pretreatment with 300 µg/ml GM (30 min) limited NADH production capacity in apical and basal turn I/OHCs (Fig. 4A, B). Although NADH Fl in apical turn I/OHCs was significantly decreased by GM, GM-pretreated apical I/OHCs were similar to controls after 30 min. In contrast, NADH Fl in basal turn I/OHCs was significantly limited throughout the NaCN exposure period (p<0.001). Suppression of NADH Fl after GM exposure was similar for apical turn low-frequency I/OHCs (Fig. 4C). In contrast, suppression of NADH Fl was greater in GM-exposed, basal turn high-frequency OHCs than in basal turn IHCs (Fig. 4D, p<0.01) during the initial 20 min of the NaCN exposure period.

### GM Suppresses NADH Fl via Krebs Cycle Inhibition

**Figure 5:**
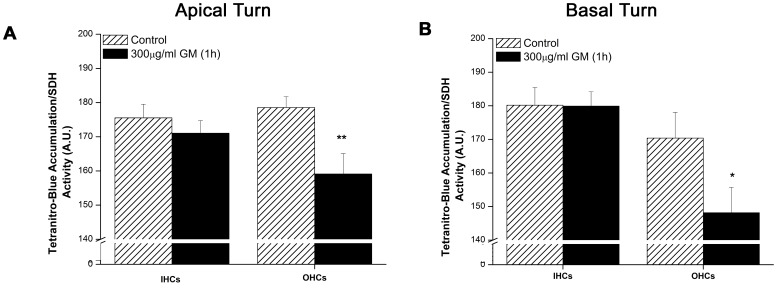
Acute GM exposure decreased succinate dehydrogenase activity OHCs. A) Apical turn OHCs display significant decreases in SDH activity during acute GM exposure, while apical turn IHCs do not (n_GM_ = 9, n_Cont_ = 8). B) Basal turn OHCs, not IHCs, display significant decreases in SDH activity during GM exposure (n_GM_ = 9, n_Cont_ = 8). * = p<0.05, ** = p<0.01.

Succinate dehydrogenase (SDH), the only dual-role enzyme in mitochondrial metabolism, participates in both the Krebs cycle (NADH reduction) and electron transport chain (NADH oxidation). With subunits A and B facing the mitochondrial matrix and subunits C and D bound to inner mitochondrial membrane, SDH couples the oxidation of succinate to fumarate in the Krebs cycle and the reduction of ubiquinone to ubiquinol in the electron transport chain [37]. SDH histochemistry is a long standing, semi-quantitative method for determining if cochlear I/OHC are metabolically compromised [31]. As a second assessment of GM’s capacity to decrease NADH production, Krebs cycle activity, indicated by SDH activity, was measured in cochlear I/OHCs after acute GM exposure (1 h, 300 µg/ml, T1 buffer). In apical turn, low-frequency regions of the cochlea, IHCs maintained SDH activity after GM treatment. In apical OHCs SDH activity was significantly decreased by GM (OHCs_control_ = 178.55±3.21, OHCs_GM_ = 159.11±5.947 arbitrary units, t(15) = 2.659, p<0.01, Fig. 5A). An OHC-specific decrease in SDH activity was also observed in basal turn, high-frequency regions of the cochlea (OHCs_control_ = 170.39±7.652, OHCs_GM_ = 148.15±7.56 A.U., t(15) = 2.019, p<0.05, Fig. 5B).

## Discussion

Examination of baseline NADH Fl intensities in high- and low-frequency I/OHCs revealed endogenous differences in I/OHC mitochondrial metabolism. Specifically, OHC NADH Fl was greater than IHC NADH Fl in both apical and basal regions of the cochlea (Fig. 1A,B). The largest difference in baseline NADH Fl occurred between basal turn IHCs and OHCs. Since baseline NADH Fl represents the balance between NADH reduction and oxidation, absolute differences in NADH Fl during maximum NADH oxidation and reduction were also measured. Absolute changes in NADH Fl in basal turn OHCs were significantly larger than the changes observed in basal turn IHCs. Relative changes in NADH redox state were similar for I/OHCs in both apical and basal regions of the cochlea. For the purposes of the current report, the I/OHC relative redox scale represents the maximum range of NADH Fl capable of occurring in cochlear HCs. This scale will be used to assess the relative oxidative and reductive impact of elevated glucose and GM on I/OHC mitochondrial metabolism.

The current report shows basal turn, high-frequency OHCs are metabolically responsive to GM and elevated glucose concentrations while high-frequency IHCs and low-frequency I/OHCs are substantially less sensitive. Basal turn OHCs displayed a robust increase in NADH Fl when exposed to high glucose, Krebs cycle substrate modified media (Fig. 3A, B). Likewise, the GM NADH effect was preferentially observed in basal turn OHCs (Fig. 2A, B). This metabolic predisposition may bias basal turn OHC responses to a variety of cochlear insults, particularly those directly involving energy metabolism and ROS production.

GM was shown to rapidly and preferentially inhibit mitochondrial metabolism in basal turn, high-frequency OHCs (Fig. 2A, B). The observed decrease in mitochondrial function is consistent with the well-known enhanced susceptibility of high-frequency basal turn OHCs to preferentially undergo apoptosis after AG exposure. In light of the relative redox scale of maximum NADH oxidation (∼30% decrease in NADH Fl) in basal turn OHCs, the ∼12% decrease in NADH caused by GM suggests that up to a third of the utilizable NADH pool was oxidized during GM exposure. This basal turn, OHC-specific effect suggests high-frequency OHCs may undergo as much as a 33% decrease in ATP production during acute GM treatment while ATP production in apical turn I/OHCs and basal turn IHCs is relatively undisturbed. This high-frequency OHC-specific decrease in NADH, and presumably ATP production capacity, is consistent with other studies indicating diminished ATP availability triggers apoptosis while large-scale losses of ATP trigger necrosis in a variety of cell types [25], [26], [38]. Given that ototoxic HC death is frequently attributed to apoptosis [39]–[42] rather than necrosis or caspase-independent mechanisms [7], [43], the decrease in NADH Fl described in the current report is consistent with a moderate decline in ATP production capacity subsequent commitment of cochlear HCs to undergo apoptosis.

Although one other report has described a rapid decrease in OHC metabolism during acute GM exposure [34], this is the first report to describe a mechanism for the observed decrease in NADH Fl. As previously mentioned, steady-state NADH Fl represents the net activities of two opposing processes; Krebs cycle-mediated NADH reduction and electron transport chain-mediated NADH oxidation. T2 buffer-produced a 10–15% increase in NADH Fl in basal turn OHCs which approached the maximum NADH reduction capacity for basal turn OHCs (∼ 20%). Surprisingly, the GM NADH effect remained undeterred. Furthermore, apical turn I/OHCs, which failed to display significant GM-induced changes in NADH Fl in T1 buffer (baseline), displayed transient, yet significant, decreases in NADH Fl when exposed to GM in T2 buffer (Fig. 3A). The observed T2-mediated increase in NADH production in apical turn I/OHCs indicates apical low-frequency HCs assumed activated metabolic profiles similar to those observed in high-frequency OHCs. As a result, the GM NADH effect was only observed in apical turn I/OHCs bathed in T2 buffer.

These findings also suggest that, if apical turn I/OHCs assume activated high-frequency OHC-like metabolic profiles, GM-triggered ROS production in apical turn I/OHCs would be enhanced. Specifically, the coupling of oxidative phosphorylation and ATP synthesis is not absolute. During normal respiration, 2–3% of the oxygen utilized by a given cell will form ROS after electrons prematurely exit the electron transport chain [44]. When the NADH/NAD^+^ ratio and/or the reduced/oxidized ubiquinone pool are altered, the fidelity of ‘appropriate’ electron transfer decreases resulting in ROS production [45]–[47].

The GM-NADH effect was essentially doubled in high-frequency basal turn OHCs bathed in T2 buffer (Fig. 3D), relative to T1 buffer (Fig. 2B). If maximum NADH oxidation causes a ∼30% decrease in NADH Fl in basal turn OHCs, the observed ∼20% GM-induced decrease in NADH in high-frequency OHCs bathed in T2 buffer suggests nearly two thirds of the utilizable NADH pool may be oxidized during GM exposure. This also suggests a large decrease in ADP phosphorylation potential and consequently ATP production capacity in OHCs. As indicated above, ROS production is predicted to also increase in T2-bathed, relative to T1-bathed high-frequency I/OHCs. The appearance of the GM NADH effect in low-frequency I/OHCs and enhanced GM NADH effect observed in basal turn OHCs suggests increasing NADH production acutely promotes compensatory increases in NADH oxidation. Akin to the difference in distances required to stop a slow-moving or rapidly-moving car, GM-induced decreases in NADH production capacity would produce a larger, yet transient decrease in NADH in I/OHCs with elevated baseline NADH reduction and oxidation.

To determine if the GM NADH effect is caused by diminished NADH reduction, NADH production capacity was evaluated. Given that NaCN inhibits NADH oxidation, NADH production capacity, or the amount of NADH capable of being produced at any given time can be assessed. NADH production capacity was diminished in I/OHCs pretreated with GM (Fig. 4). Although NADH production capacity was significantly decreased in apical and basal turn I/OHCs, a profound and prolonged decrease in NADH production capacity was observed in high-frequency OHCs. Notably, NADH production capacity in GM-treated apical turn I/OHCs reached control levels after 30 min. I/OHCs also maintained similar NADH levels throughout the experiment. In stark contrast, high-frequency, basal turn OHCs displayed a robust decrease in NADH production capacity after GM treatment. Unlike low-frequency I/OHCs, high-frequency I/OHCs did not recover from GM-induced decreases in NADH production capacity. In concert, the above experiments clearly indicate a GM-induced decrease in NADH production capacity occurs within minutes of GM application. Furthermore high-frequency OHCs, known to be preferentially damaged by GM, display the greatest decline in NADH and NADH production capacity. To this end, the GM NADH effect is not likely to be due to an increase in NADH oxidation. However, this is difficult to confirm when NADH reduction is compromised. Further studies will need to be performed to confirm this observation.

As an additional verification of the observed GM-induced decrease in NADH production capacity, SDH activity was measured in GM-treated cochlear I/OHCs. In light of the fact that SDH participates in both the Krebs cycle and electron transport chain, assays of SDH activity assays must be cautiously interpreted. Although the activity of other enzymes in the Krebs cycle can be measured (citrate synthase, aconitase, to name just two), SDH is the only Krebs cycle enzyme that can be assayed in intact tissues. Likewise, accurate measurements of cell-specific oxygen consumption, an additional method for measuring metabolic activity, are difficult in heterogeneous cell populations. Recall that the current study investigates metabolic differences between cochlear I/OHCs. Given that 1) cochlear I/OHCs reside within the cochlear partition which contains both sensory and supporting cells, 2) OHCs are nearly three times more numerous than IHCs (see Fig. 1C,D) and 3) there is no way to isolate large quantities of unperturbed IHCs and OHCs for IHC- and OHC-specific mitochondrial analyses, SDH activity is the best assay for IHC- and OHC-specific Krebs cycle analyses. Likewise, a recent, extensive review of the actions of SDH indicates SDH/Complex II of the mitochondrial respiratory system is ‘the central mediator’ of most pathologies involving oxidative damage [45]. Akin to the observed GM-induced alterations in NADH, SDH activity is also shown to modulate NADH/NAD^+^ ratios in multiple cell types.

In the current report, acute GM exposures decreased SDH activity in OHCs (Fig. 4). Although others have shown that longer GM exposures (≥4 h) decrease SDH activity in I/OHCs [48], this is the first report to show GM rapidly (1 h) decreases SDH activity. When the observed decrease in SDH activity is considered in conjunction with GM-induced decreases in NADH Fl intensity, a GM-induced deficit in NADH reduction is indicated. One function of SDH is to donate electrons, from succinate, to the ubiquinone pool in the electron transport chain. NADH also donates electrons to of the electron transport chain (complex I). Therefore, NADH could continue to donate electrons to the electron transport chain in the absence of succinate oxidation. Recall, however, that NADH is the primary reducing equivalent generated in the Krebs cycle. If SDH is no longer capable of oxidizing succinate to fumarate, NADH production and Krebs cycle activity would be significantly impeded. Given that the amount of NADH generated during β-oxidation and glycolysis or regenerated via the malate-aspartate shuttle is significantly less than Krebs cycle-produced NADH [49], a net decrease in NADH would occur when SDH activity is decreased. As such NADH oxidation (complex I, electron transport chain) could continue while NADH reduction (Krebs cycle) is reduced. Once the NADH pool is exhausted, as indicted by the net decrease in NADH Fl, electron transport dysfunction and decreased ATP production would ensue.

A number of groups have shown a decrease in HC viability, cytochrome c release, AIF release, and mitochondrial permeability transition pore (MPTP) opening using similar AG exposures (recent reviews [50]–[53]). Of particular importance, Dehne et al. [16] observed opening of the MPTP in cochlear outer (not inner) HCs required ≥4 h of exposure to GM at considerably higher concentrations (500–1000 µM) than what was used in the current report (300 µg/ml ∼ 150 µM). Furthermore, cyclosporin A, a MPTP inhibitor, did not significantly increase OHC survival within this time frame, although an increase was observed. Given that rapid opening of the MPTP would cause a decrease in NADH Fl, the 300 µg/ml GM dose used in the current study was purposely chosen to 1) be closer to a physiologically relevant range and 2) avoid rapid opening of the MPTP. MPTP opening, an event that is certainly not requisite for excess ROS production, is the end result of gross perturbations in mitochondrial metabolism. As such, we do not predict opening of the MPTP in the acute GM exposures utilized in the current report. Rather the current studies describe how AGs *directly* and differentially alter IHC and OHC mitochondrial metabolism in the absence of MPTP opening. To this end, it is fully anticipated that sustained GM-induced alterations NADH metabolism (>4 h) would reduce hair cell viability, increase the probability of MPTP opening and trigger the release of mitochondrial-specific pro-apoptotic signaling factors as observed in other cell populations [54]. Indeed, our findings are also consistent with the work of Alharazneh et al. [13], who showed that exposure to 0.25 mM GM for 1 h followed by a 48 h recovery period resulted in a tonotopic loss of OHCs in the basal and middle turns while HC loss was negligible immediately following GM exposure. When considered in conjunction with the results described in the current study, 1 h of exposure to GM (250–300 µg/ml) preferentially produces metabolic dysfunction in high-frequency, basal turn OHCs that appear to be sufficient to trigger a pro-apoptotic cascade of events which ultimately lead to the demise of high-frequency OHCs.

Given that the density of functional mitochondria (Fig. 1E) is greater in OHCs than IHCs regardless of cochlear location, elevated baseline NADH Fl may be due to differences mitochondrial densities. This explanation does not, however, seem sufficient to explain why 1) T2 buffer preferentially enhanced NADH Fl in high-frequency, not low-frequency OHCs, 2) why low-frequency I/OHCs exhibit similar responses to GM and, 3) why the GM-NADH effect was greatest in high-frequency, basal turn OHCs. These results indicate the endogenous metabolic profiles of I/OHCs are fundamentally different. As such, high-frequency basal turn OHCs appear to be metabolically biased to rapidly respond to alterations in their microenvironment including increased glucose and GM exposure. This report identifies endogenous differences in I/OHC NADH metabolism and a GM-mediated decrease in NADH production capacity and Krebs cycle activity within minutes of GM application. Multiple ROS production mechanisms have been proposed in studies using prolonged AG exposures (≥24 h) [55]–[57]. Given that AGs have been shown to alter mitochondrial protein synthesis [14], [15], any study of AG ototoxicity must differentiate between immediate and long-term alterations in mitochondrial function. Of particular significance, the results described herein occur on a timescale too short to include GM-induced changes in mitochondrial protein synthesis and turnover. Instead these results strongly suggest GM *directly* alters mitochondrial function in cochlear I/OHCs.

Although SDH and NADH are involved, the exact nature of this interaction awaits further study. As such, the novel and intriguing findings of the current report will undoubtedly function as a catalyst for future studies aimed at deciphering the true mechanism(s) by which GM disrupts mitochondrial metabolism and presumably triggers mitochondrial ROS production. Notably, the NOX family of NADPH oxidases is another recognized source of ROS in cochlear HCs [58]–[60]. NOX-mediated redox signaling is, however, unlikely to trigger ROS production during the early stages of GM exposure described in the current report. Specifically, several groups have shown NOX-dependent ROS production in HCs requires NOX activation, a process requiring over 6 h to trigger significant increases in ROS [58], [60], [61]. Therefore, the endogenous metabolic biases observed in the current report indicate AG-mediated ROS production in cochlear HCs is likely the result of direct mitochondrial alterations and rapid metabolic dysfunction.

Given that AG-induced ROS production is a well-documented trigger for AG ototoxicity and irreversible HC loss (recent reviews [50], [55]) and decreased NADH levels are associated with profound mitochondrial ROS production (recent review [45]), the current study highlights a need to further elucidate the role(s) mitochondria in mediating AG ototoxicity and, potentially, numerous other HL pathologies including ARHL and NIHL.

## Materials and Methods

### Cochlear Explants

All experiments were performed using acutely-cultured, intact cochlear (organ of Corti) explants. Cochleae obtained from CO_2_ asphyxiated postnatal day 6 (P6±1d) FVB mice were dissected in HEPES-buffered L-15. Intact explants were acutely cultured (24–30 h) at 37°C and 5% CO_2_ in Dulbecco’s modified Eagle’s Medium/F12 medium (Invitrogen, Carlsbad, CA., USA) supplemented with 10% FBS (Invitrogen). The number of cochlear explants examined in each experiment and condition is indicated in the respective figure caption. Cochlear preparations with intact, viable HCs were maintained at 32±4°C in one of two modified Tyrodes buffers (see below) throughout imaging. During each experiment I/OHCs located in apical, low-frequency (20% of cochlear length) and/or basal, high-frequency (80% of cochlear length) regions were compared. Unless otherwise noted, reagents and solutions were obtained from Sigma-Aldrich (St. Louis, MO., USA). Animal care and use procedures were approved by the Creighton University Animal Care and Use Committee.

### Imaging Methods

Baseline modified Tyrodes imaging buffer (T1) contained 5 mM glucose, 135 mM NaCl, 5 mM KCl, 1 mM MgCl_2_, 1.8 mM CaCl_2_ and 20 mM HEPES. Enhanced modified Tyrodes imaging buffer (T2) was generated by adding Krebs cycle substrates (3 mM glutamate, 2 mM pyruvate) and increasing the glucose content of T1 to 10 mM. Each solution was adjusted to 310±5 mOsm and a pH of 7.35±0.05.

Endogenous NADH was excited using femtosecond pulses of 740 nm light from a MaiTai DeepSee laser (Newport, Irvine, CA, USA) using an upright Zeiss LSM 510 META NLO scanning confocal microscope and a 60×, 0.9 N.A. water immersion lens (Olympus, Center Valley, PA, USA). Successive focal planes (2.5 µm apart) were imaged through each cochlear preparation. Z-stack image series were obtained at 10 min intervals. Non-descanned NADH Fl was collected using a 500 nm long pass dichroic mirror (500 DCXR, Chroma, Bellows Falls, VT, USA) and 460/80 bandpass (BP) filter. During NADH imaging, one baseline image was obtained before application of 300 µg/ml GM.

### Assays of Metabolic Activity

Endogenous, steady-state NADH Fl intensities were obtained by imaging apical and basal turn I/OHCs under identical imaging parameters using T1 buffer. To determine if absolute and/or relative differences in I/OHC NADH oxidation and reduction occur, 10 µM Carbonyl cyanide-*p*-trifluoromethoxyphenylhydrazone (FCCP) or 10 µM sodium cyanide (NaCN) were administered, respectively. Others have shown these concentrations are sufficient to cause maximum NADH reduction and oxidation in cochlear HCs [35]. NADH Fl changes, calculated as the difference in NADH Fl before and 10 min after each treatment, were compared in apical, low-frequency and basal, high-frequency regions of the cochlea. NaCN was also used to calculate NADH production capacity before and after 300 µg/ml GM. By specifically inhibiting cytochrome C oxidase (mitochondrial complex IV), NaCN prevents NADH oxidation while Krebs cycle-mediated NADH production remains active. The net result is an increase in the NADH/NAD^+^ ratio indicated by an increase in NADH Fl. Given that NADH oxidation (conversion to non-Fl NAD^+^) is inhibited, NaCN-induced changes in NADH Fl are indicative of the total amount of NADH produced, or the NADH production capacity for each cell. NaCN was applied 30 min after 300 µg/ml GM.

Given that cochlear I/OHCs are 1) surrounded by supporting cells in the cochlear partition and 2) are extremely difficult to individually isolate in sufficient quantities to perform cell type specific analyses of metabolic activity, options for measuring metabolic activity are limited to those applicable to intact cells. Succinate dehydrogenase (SDH) histochemistry is a long standing technique used to measure metabolic activity and HC viability [28]–[30], [62]–[66]. SDH histochemistry performed similarly to previous reports [29], [67], was used to semi-quantitatively measure SDH/Krebs cycle activity in control and GM exposed (1 h) cochlear preparations in conjunction with NADH Fl changes. Cochlear preparations were exposed to a SDH staining solution containing 1∶1:2 parts of 0.2 M sodium succinate, 0.2 M phosphate buffered saline (pH 7.6), 0.1% tetranitro-blue tetrazolium, respectively. After 45 min of exposure to the SDH staining solution at 37°C, cochlear cultures were fixed in 10% formalin for 2 h. Transmitted light images of the insoluble tetranitro-blue precipitate, indicative of SDH activity, were obtained using the transmitted light detector of the Zeiss confocal microscope previously described.

Functional mitochondria were labeled with Mitotracker Red CM-H_2_XRos (Invitrogen). Briefly, cochlear explants were incubated in 200 nM Mitotracker Red in T1 buffer for 10 min at 37°C and 5% CO_2_. Next, cochlear explants were rinsed and immediately imaged under identical conditions in T1 buffer. Images were acquired using 543 nm as the excitation wavelength and collected using a 565–615 nm emission filter. Relative differences in Mitotracker Red Fl were calculated and compared.

### Image Analysis

As previously mentioned, successive focal planes (2.5 µm apart) were collected throughout each preparation. To compensate for differences in I/OHC volume, average NADH and Mitotracker Red Fl in each I/OHC was calculated by measuring the respective Fl in each consecutive focal plane. Individual values were averaged to determine HC-specific Fl intensities. These intensities were pooled to determine the mean I/OHC values for each cochlear preparation. Individual transmitted light images of nitro-blue tetrazolium accumulation were analyzed to determine SDH activity in I/OHCs. Raw pixel data was analyzed using Image J [68] and evaluated using Students t-test analyses performed in Excel. Data were plotted using OriginLab (Northampton, MA, USA).
